# Kaempferol enhances cisplatin's effect on ovarian cancer cells through promoting apoptosis caused by down regulation of cMyc

**DOI:** 10.1186/1475-2867-10-16

**Published:** 2010-05-11

**Authors:** Haitao Luo, Matthew K Daddysman, Gary O Rankin, Bing-Hua Jiang, Yi C Chen

**Affiliations:** 1 Natural Science Division, Alderson-Broaddus College, Philippi, WV, 26416, USA; 2 Mary Babb Randolph Cancer Center, Department of Microbiology, Immunology, and Cell Biology, West Virginia University, Morgantown, WV, USA; 3 Department of Pharmacology, Physiology and Toxicology, Joan C. Edwards School of Medicine, Marshall University, Huntington, WV, USA

## Abstract

**Background:**

Ovarian cancer is one of the most significant malignancies in the western world. Studies showed that Ovarian cancers tend to grow resistance to cisplatin treatment. Therefore, new approaches are needed in ovarian cancer treatment. Kaempferol is a dietary flavonoid that is widely distributed in fruits and vegetables, and epidemiology studies have revealed a protective effect of kaempferol against ovarian cancer risk. Our early studies also found that kaempferol is effective in reducing vascular endothelial growth factor (VEGF) expression in ovarian cancer cells. In this study, we investigated kaempferol's effects on sensitizing ovarian cancer cell growth in response to cisplatin treatment.

**Results:**

Ten chemicals were screened for sensitizing OVCAR-3 ovarian cancer cell growth in response to cisplatin treatment. For kaempferol, which shows a significant synergistic interaction with cisplatin, expression of ABCC1, ABCC5, ABCC6, NFkB1, cMyc, and CDKN1A genes was further examined. For cisplatin/kaempferol treatments on OVCAR-3 cancer cells, the mRNA levels of ABCC1, ABCC5, and NFkB1 did not change. However, significant inhibition of ABCC6 and cMyc mRNA levels was observed for the cisplatin/kaempferol combined treatment. The CDKN1A mRNA levels were significantly up-regulated by cisplatin/kaempferol treatment. A plot of CDKN1A mRNA levels against that of cMyc gene further revealed a reverse, linear relationship, proving cMyc's regulation on CDKN1A gene expressions. Our work found that kaempferol works synergistically with cisplatin in inhibiting ovarian cancer cell viability, and their inhibition on cell viabilities was induced through inhibiting ABCC6 and cMyc gene transcription. Apoptosis assay showed the addition of 20 μM kaempferol to the cisplatin treatment induces the apoptosis of the cancer cells.

**Conclusions:**

Kaempferol enhances the effect of cisplatin through down regulation of cMyc in promoting apoptosis of ovarian cancer cells. As a dietary component, kaempferol sensitizes ovarian cancer cells to cisplatin treatment and deserves further studies for possible applications in chemotherapy of ovarian cancers.

## Background

Ovarian cancer is one of the most important diseases for women in Western countries. It is the fifth leading cause of cancer-related deaths [1]. Treatment of ovarian cancers usually involves surgery and chemotherapy. The combination of cisplatin and paclitaxel as a chemotherapeutic regimen has improved the survival of ovarian cancer patients. However, the results are not satisfying because of drug resistance developed by cancer cells [2]. The cancer frequently progresses after the treatment and the majority of ovarian cancer patients die as cancer later relapses [3]. Therefore, it is important to identify new approaches for the treatment of ovarian cancer.

Flavonoids are polyphenolic natural compounds which are present in a wide variety of fruits and vegetables [4] and are protective against some forms of cancer [5]. It has been reported that dietary flavonoids reduce the risks of humans to cardiovascular disease [6], prostate cancer [7], colorectal cancer [8], and renal cancer [4]. Flavonoids were also reported to inhibit cell growth and proliferation [9] and induce cell toxicity [10] in cancer cells.

Cisplatin (cis-diamminedichloroplatinum(II)) is commonly used in the treatment of various cancers. Cisplatin is activate inside the cell and reacts with guanine residues in DNA. The binding of cisplatin to DNA changes the secondary structure of DNA and consequently the metabolism of the cell. The exact mechanism by which cisplatin influences the metabolism of the cell and consequently cell growth is unclear [11].

Several genes have been reported to potentially play a role in cisplatin resistance. The ABCC1, ABCC5, and ABCC6 genes are members of the ABCC family of membrane transport proteins. These genes have been implicated in drug resistance of a variety of anticancer drugs including platinum based drugs such as cisplatin. Inhibition of these genes is ideal to reduce the efflux of anticancer drugs out of the cell [12]. The NFκB1 gene is a subunit of the NFκB gene, an important regulator of genes controlling a variety of cell survival process including proliferation and apoptosis. Activation of the NFκB gene has been implicated in many human cancers [13]. The genes cMyc and CDKN1A are also important regulators of cell proliferation and apoptosis. However, their role in cisplatin resistance is unclear.

In this study, we investigated the sensitization effects of eight flavonoids (luteolin, genistein, quercetin, kaempferol, taxifolin, rutin hydrate, naringin, and apigenin), and two forms of vitamin E (tocopherol and tocopherol succinate) on the cisplatin induced killing of cells in the ovarian cancer cell line OVCAR-3. To measure the sensitization effect we used a novel statistical model to distinguish between true sensitization of the cells to cisplatin and the additive effect of the combined toxicity of the sensitization chemical and cisplatin. For any chemicals that showed a sensitization effect we further measured the effect of the cisplatin-chemical combination on the expression of ABCC1, ABCC5, ABCC6, NFκB1, cMyc and CDKN1A genes.

## Results

### Kaempferol synergistically enhances cisplatin's effect on inhibiting proliferation of OVCAR-3 cancer cells

**Figure 1:**
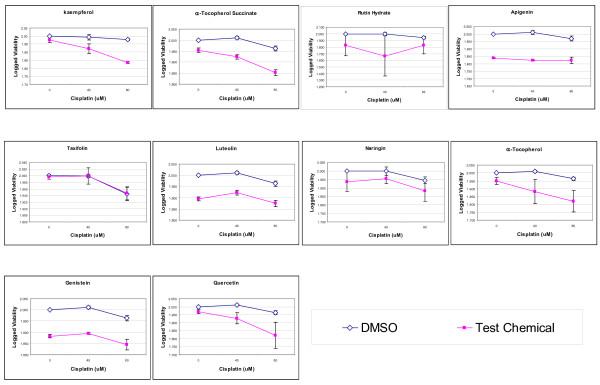
**Effect on cell proliferation in OVCAR-3 cancer cells**. OVCAR-3 cells (8 × 10^3^) were seeded into wells in 96-well cell culture microplates, incubated for 16 hours, and treated with 0, 40, and 80-μM Cisplatin with or without other chemicals of 20 μM for 24 hours in triplicates. Cell viability was analyzed with MTS-based assay, normalized as percent of control, logged and fitted to linear regression models to check cisplatin and other chemicals' effects on cell proliferation, and their interactions. Data represents MEAN ± SE from 3 independent experiments.

All 10 chemicals, including 8 flavonoids and 2 α-tocopherols, were tested at 20-μM concentration for their additive or synergistic effects with cisplatin on OVCAR-3 cancer cells. As shown in Figure 1, OVCAR-3 cancer cells are relatively resistant to cisplatin treatment, remaining 88.1-95.3% (or 1.945-1.979 when logged) viability at 80-μM cisplatin concentration. The effects of test chemicals alone (at 20-μM) vary from no effect (99% for taxifolin) to moderate effect (67% for rutin hydrate). With the exception of taxifolin, co-treatment with cisplatin and test chemicals was more effective inhibiting cancer cell proliferation than cisplatin alone, although the majority of these combined effects are additive (p > 0.05 for coefficients of Cisplatin*Drug, Table 1). However, for kaempferol and α-tocopherol succinate, the resistance to cisplatin in OVCAR-3 cells was reduced, with the cell viability down-regulated to 68.4% (1.835 when logged) and 71.3% (1.853 when logged), respectively. The slope of cisplatin treatment, by linear regression analysis, is changed from -0.012 to -0.070 by co-treatment with 20-μM kaempferol, and from -0.019 to -0.051 by co-treatment with 20-μM α-tocopherol succinate, demonstrating a significant sensitization (p = 0.001 for kaempferol and 0.015 for α-tocopherol succinate). The goodness of fit (R^2 ^= 0.901 for kaempferol and 0.891 for α-tocopherol succinate) in these models suggests a successful regression on logged viability data with kaempferol and α-tocopherol succinate co-treatments (Table 1).

**Table 1:** Linear regression to analyze cisplatin and/or compound's effect on OVCAR-3 cell proliferation.

	Kaempferol		α-Tocopherol Succinate		Rutin Hydrate	
R2	0.901		0.891		0.188	
	**B**	**Sig.**	**B**	**Sig.**	**B**	**Sig.**
Constant	2.002	0.000	2.010	0.000	2.009	0.000
Cisplatin	-0.012	0.198	-0.019	0.040	-0.028	0.789
Compound	-0.022	0.238	-0.048	0.006	-0.237	0.221
Cispl. × Comp.	-0.058	**0.001**	-0.032	**0.015**	0.029	0.844

						

	**Apigenin**		**Taxifolin**		**Luteolin**	

R2	0.947		0.415		0.841	
	**B**	**Sig.**	**B**	**Sig.**	**B**	**Sig.**
Constant	2.008	0.000	2.009	0.000	2.010	0.000
Cisplatin	-0.015	0.119	-0.028	0.035	-0.019	0.076
Compound	-0.173	0.000	-0.003	0.902	-0.102	0.000
Cispl. × Comp.	0.0080	0.571	0.003	0.884	0.008	0.548

						

	**Naringin**		**α-Tocopherol**		**Genistein**	

R2	0.296		0.541		0.885	
	**B**	**Sig.**	**B**	**Sig.**	**B**	**Sig.**
Constant	2.009	0.000	2.010	0.000	2.010	0.000
Cisplatin	-0.028	0.306	-0.019	0.521	-0.019	0.086
Compound	-0.058	0.243	-0.062	0.253	-0.117	0.000
Cispl. × Comp.	0.001	0.989	-0.046	0.269	0.0001	0.994

						
				
	**Quercetin**					
				
R2	0.567					
	**B**	**Sig.**				
Constant	2.010	0.000				
Cisplatin	-0.019	0.467				
Compound	-0.029	0.533				
Cispl. × Comp.	-0.056	0.134				

Data from 3 independent experiments were modeled as (Viability = Constant + Cisplatin + Compound + Cisplatin* Compound) to analyze effects of Cisplatin, Compound, and Cisplatin-Compound interactions. Tabulated are standardized coefficients (B) with corresponding p values (Sig.) for each model. The goodness of fit, R^2^, is also listed for each model.

### Kaempferol enhances cisplatin's effect on gene transcription for ABCC6, cMyc and CDKN1A in OVCAR-3 cancer cells

**Figure 2:**
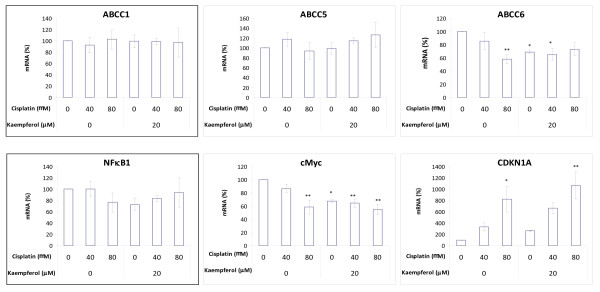
**Kaempferol and/or cisplatin's effect on ABCC1, ABCC5, ABCC6, NFκB1, cMyc and CDKN1A mRNA levels**. OVCAR-3 cells (5 × 10^5^) were seeded in 60-mm cell culture dishes and incubated for 16 hours before chemical treatment (0, 40, and 80-μM cisplatin with or without 20-μM kaempferol) for another 24 hours. RNA was extracted with TRIzol Reagent and quantitated by qRT-PCR as described in Materials and Methods. Gene mRNA levels were adjusted by GAPDH and expressed as percent of control. Data represents MEAN ± SE from 3 independent experiments. * *p *< 0.05 as compared to control by ANOVA Dunnett t test. ** *p *< 0.01 as compared to control by ANOVA Dunnett t test.

**Figure 3:**
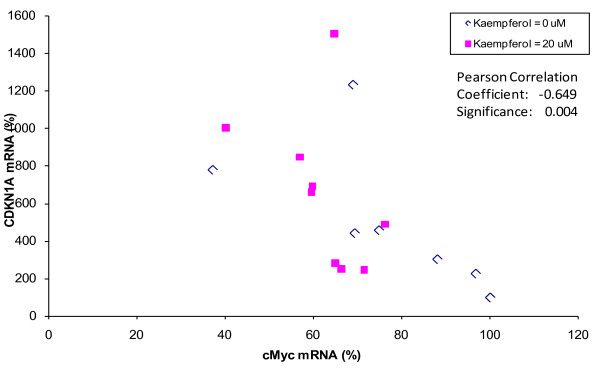
**Negative correlation between cMyc and CDKN1A gene mRNA levels**. The mRNA levels of CDKN1A and cMyc from 3 independent experiments were expressed as percent and plotted against each other for Pearson Correlation analysis. A strong negative association exits for CDKN1A and cMyc genes (as regulated by cisplatin treatment) regardless of kaempferol treatment.

Because kaempferol sensitizes OVCAR-3 cancer cells' response to cisplatin treatment, the effect on gene expression by cisplatin and/or kaempferol was further evaluated by analyzing mRNA levels of ABCC1, ABCC5, ABCC6, NFκB1, cMyc and CDKN1A genes. As shown in Figure 2, cisplatin treatment, with or without kaempferol, does not alter mRNA levels significantly for ABCC1, ABCC5, and NFκB1 genes. However, cisplatin decreased ABCC6 and cMyc mRNA levels in a dose-dependent manner, with a remaining mRNA level of about 58% at 80-μM concentration (p < 0.05). Kaempferol treatment also inhibited ABCC6 and cMyc genes transcription down to 68% (p < 0.01). Combination of kaempferol with 40-μM cisplatin further inhibited ABCC6 and cMyc genes mRNA levels down to 65% (p < 0.05), and with 80-μM cisplatin leads to the lowest mRNA level of 55% for cMyc(p < 0.01), although no enhancement is observed for ABCC6 gene at 80-μM cisplatin concentration. For the CDKN1A gene, cisplatin increased mRNA levels dose-dependently, reaching 818.7% at 80-μM concentration (p < 0.05), while kaempferol failed to increase CDKN1A mRNA level significantly (p = 0.891). However, combination of the two chemicals brings the CDKN1A mRNA level up to 1064.7% (p < 0.01). Due to the known regulation of the CDKN1A gene expression by cMyc, the relationship between the two genes was further examined. The mRNA levels of CDKN1A were plotted against cMyc in a linear scale and a linear relationship was observed between cMyc and CDKN1A mRNA levels, regardless of kaempferol treatment (Figure 3). The two sets of data were pooled for correlation analysis, and the Pearson correlation coefficient is -0.649, demonstrating a strong negative association (p < 0.01).

### Kaempferol promotes apoptosis in OVCAR-3 cancer cells treated with cisplatin

**Figure 4:**
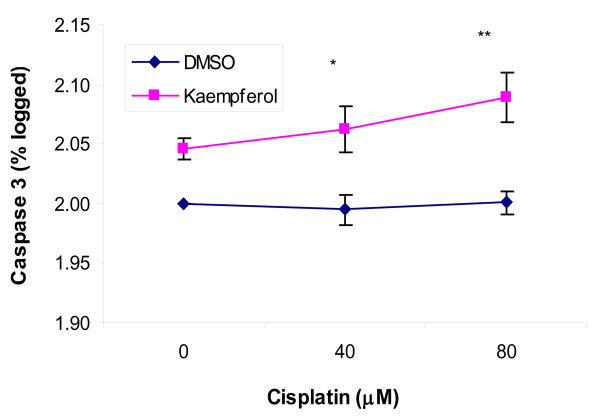
**Effects in inducing caspase-3 levels in OVCAR-3 cancer cells**. OVCAR-3 cells (2 × 10^6^) were seeded in 60-mm dishes, incubated for 16 hours, and treated with kaempferol (0 or 20 μM) and cisplatin (0, 40, and 80 μM) for 2 hours. Caspase-3 levels were determined with a colorimetric assay kit and normalized to total protein levels determined with a BCA assay kit. Normalized caspase-3 levels were expressed as percent of control, logged and fitted to linear regression models to check cisplatin and kaempferol's effects on cell apoptosis, and their interactions. Data represents MEAN ± SE from 4 independent experiments. * *p *< 0.05 as compared to control by ANOVA Dunnett t test. ** *p *< 0.01 as compared to control by ANOVA Dunnett t test.

Cisplatin and kaempferol were also tested for their effects in inducing apoptosis in OVCAR-3 cancer cells. Caspase-3 levels in OVCAR-3 cells range from 5-13 pg/μg-Total Protein (TP), but showed considerable inter-experiment variances. Caspase-3 levels were expressed as percent of control within each experiment and plotted in logarithmic scale for linear regression analysis. As shown in Figure 4, cisplatin treatment alone, up to 80 μM, does not cause any noticeable changes in caspase-3 levels as compared to the 7.4 pg/μg-TP in control (p > 0.70), while a 20-μM kaempferol treatment raised caspase-3 levels to 8.2 pg/μg-TP. More importantly, when combined with kaempferol treatment, cisplatin begins to have a dose-dependent effect in inducing caspase-3 levels, a significant sensitization proven by linear regression analysis (p < 0.05 for coefficient of Cisplatin*Kaempferol, R^2 ^= 0.905). The caspase-3 level is 8.6 pg/μg-TP for cells treated with 40-μM cisplatin and 20-μM kaempferol (p < 0.05) and 9.2 pg/μg-TP for cells treated with 80-μM cisplatin and 20-μM kaempferol, significantly higher than that of control.

## Discussion

There are many different chemotherapy agents for treating cancer. One of the most commonly used chemotherapy drugs is cisplatin. Cisplatin is commonly used in cancers of the head, neck, bladder, ovary, and testis. Unfortunately, many cancer cells are resistant to cisplatin treatment or may become resistant with treatment. Currently the only way to overcome acquired resistance to cisplatin in cancer cells is by increasing dosage, which results in higher toxicity in normal body cells [14]. Resistance to cisplatin treatment can be due to lack of the p53 gene [15], by the activation of cell survival genes such as nuclear factor-κB (NF-κB) [16], and by the reduced cellular concentration of cisplatin [14].

OVCAR-3 cells were found to be resistant to cisplatin treatment with 88.1-95.3% viability at 80-μM cisplatin concentration. Our previous studies found that flavonoids, including kaempferol, inhibit ovarian cancer cell growth and VEGF expression [17]. Some flavonoids are reported to have sensitization effects on cisplatin [15,18]. The 10 chemicals screened, varied in their viability suppression from light to moderate. Among the 10 chemicals tested only kaempferol, a flavonoid, and α-tocopherol succinate, a vitamin E derivative, significantly sensitized OVCAR-3 cells to cisplatin treatment. With the exception of taxifolin, all of the combined treatments with cisplatin and the test chemical resulted in a more effective treatment. However, all of these effects were additive except for kaempferol and α-tocopherol succinate.

To gain an insight into the mechanism of kaempferol sensitization, the mRNA levels of ABCC1, ABCC5, ABCC6, NFκB1, cMyc and CDKN1A genes were analyzed by qPCR. The levels of ABCC1, ABCC5, or NFκB1 were not influenced by cisplatin or kaempferol. With the addition of kaempferol, cisplatin treatment significantly inhibited ABCC6 and cMyc mRNA levels. Since ABCC6 is a membrane transport protein important in the reduction of intracellular accumulation of cisplatin [12], the reduced amount of ABCC6 by kaempferol prevents the removal of cisplatin from the cell leading to the killing of the cancer cell by cisplatin.

Another mechanism of sensitization of cisplatin by kaempferol is through down regulating of cMyc and up regulation of CDKN1A. The proto-oncogene cMyc is highly expressed in proliferating cells and is commonly activated in human cancers [19]. cMyc is known to down regulate the CDKN1A gene and our study showed a strong negative association between the two (Pearson correlation coefficient, -0.649, p < 0.01). The up regulation of CDKN1A usually leads to the apoptosis of the cancer cells [20]. Our apoptosis assay showed that cisplatin alone does not induce apoptosis of the ovarian cancer cells. The addition of 20 μM kaempferol to the cisplatin treatment induces the apoptosis of the cancer cells. Therefore, kaempferol enhances the effect of cisplatin through down regulation of cMyc in promoting apoptosis of ovarian cancer cells.

## Conclusions

Our work found that kaempferol works synergistically with cisplatin in inhibiting ovarian cancer cell viability, and their inhibition on cell viabilities was induced through inhibiting ABCC6 and cMyc gene transcription. Apoptosis assay showed the addition of 20 μM kaempferol to the cisplatin treatment induces the apoptosis of the cancer cells. Therefore, kaempferol enhances the effect of cisplatin through down regulation of cMyc in promoting apoptosis of ovarian cancer cells. As a dietary component, kaempferol sensitizes ovarian cancer cells to cisplatin treatment and deserves further studies for possible applications in chemotherapy of ovarian cancers.

## Methods

### Chemicals

All 11 chemicals, including cisplatin, 2 α-tocopherols, and 8 flavonoids were obtained from Sigma (St. Louis, MO). Chemicals were dissolved in DMSO to form a 20-mM stock solution which were stored at -20°C. For cell treatment, DMSO concentrations were controlled constant through various concentration and chemical combinations.

### Cell Culture

The human ovarian cancer cell line OVCAR-3 was obtained from American Type Culture Collection (Manassas, VA). Cells were cultured in RPMI 1640 medium supplemented with 4 μmol/mL glutamine, 100 units/mL penicillin, 100 μg/mL streptomycin (VWR, West Chester, PA), and 10% US-qualified fetal bovine serum (Invitrogen, Grand Island, NY) in a humidified incubator with 5% CO_2 _at 37°C.

### Cell Proliferation Assay

Chemical effects on OVCAR-3 cell proliferation were colorimetrically determined with a "CellTiter 96^® ^Aqueous One Solution Cell Proliferation Assay" kit from Promega (Madison, WI). OVCAR-3 cells (8 × 10^3^) were seeded into wells in 96-well cell culture microplates and incubated for 16 hours. Chemical treatments (0, 40, and 80-μM Cisplatin with other chemicals of 0, or 20-μM) were then applied in triplicates for another 24 hours. After removal of medium, 100 μL freshly-prepared Aqueous One Solution (MTS tetrazolium compound) was added to each well, incubated at 37°C for 2 hours, and optical density (OD) measured at 490 nm with a microplate reader (Bio-Rad, Hercules, CA). A linear standard curve was generated by seeding different numbers of cells (0 - 1 × 10^4^) at the beginning and used to quantify chemical-treated cells' viability. Cell viability was expressed as percent of control (0-μM cisplatin and 0-μM other chemicals), and averaged from replicates. Data from 3 independent experiments were pooled for statistical analysis.

### Quantification of mRNA levels for ABCC1, ABCC5, ABCC6, NFκB1, cMyc and CDKN1A genes

Effect on transcription of the 6 genes' were determined by quantitative reverse-transcription Polymerase Chain Reaction (qRT-PCR). OVCAR-3 cells (5 × 10^5^) were seeded in 60-mm cell culture dishes and incubated for 16 hours before chemical treatment (0, 40, and 80-μM cisplatin with or without 20-μM kaempferol) for another 24 hours. Cells were washed twice with PBS (Phosphate Buffered Saline), and RNA was extracted with TRIzol Reagent (Invitrogen, Grand Island, NY). RNA was resuspended in DEPC (diethyl pyrocarbonate)-treated water and introduced to reverse transcription with oligo-dT and AMV Reverse Transcriptase from Promega (Madison, WI). Real-time PCR was deployed to amplify cDNA for the 6 genes with RT^2 ^SYBR Green qPCR Master Mix (SuperArray Bioscience, Frederick, MD) and a Chromo4 real-time detector coupled to a DNA Engine thermal cycler (Bia-Rad, Hercules, CA). Primers for CDKN1A were designed from the Primer3 website http://frodo.wi.mit.edu/primer3/ to amplify both transcript variants, and primers for other genes were chosen from the PrimerBank website http://pga.mgh.harvard.edu/primerbank/ (Table 2). The PCR program was set as follow: 95°C 10'; (95°C 20", 58°C 45", 72°C 20", 77°C 1", read plate) × 50; 72°C 5'; 58°C 1'; melting curve (65°C -95°C by 0.5°C increments). A standard curve was generated from series dilutions of PCR products to monitor amplification efficiency, and to relatively quantify samples for every gene. RNA samples without reverse transcription served as non-reverse-transcription (-RT) controls, and melting curves were checked for uniform amplification. In processing amplification curves, baselines were set as average levels through cycle 2-12, and threshold values was set as 0.01. Gene expression levels, as relatively quantified by individual standard curves, were further adjusted by GAPDH (Glyceraldehyde 3-phosphate dehydrogenase) levels, and expressed as percent of control (0-μM cisplatin and 0-μM kaempferol). Data from 3 independent experiments were pooled for statistical analysis.

**Table 2:** Primer sequences for genes analyzed.

Gene	GenBank Accession	Primers	Amplicon size
**GAPDH**	NM_002046	**CAT GAG AAG TAT GAC AAC AGC CT**	**113**
		AGT CCT TCC ACG ATA CCA AAG T	
**ABCC1**	NM_004996	**CCA GTG GGG ATC GGA CAG A**	**124**
		AGG GGA TCA TCG AAG AGG TAA AT	
**ABCC5**	NM_005688	**CCA GGG TCC TGA TTC GGG A**	**122**
		CTC CTG CCC TTT ATT GTA GGC	
**ABCC6**	NM_001171	**AGC CAG CTA CTC GTC TGT CT**	**108**
		CGA GCA TTG TTC TGA GCC A	
**NFkB1**	NM_003998	**ATT TGA AAC ACT GGA AGC ACG A**	**145**
		GCG GAT TAG CTC TTT TTC CCG	
**cMyc**	NM_002467	**TCG GAA GGA CTA TCC TGC TG**	**133**
		GTG TGT TCG CCT CTT GAC ATT	
**CDKN1A**	NM_000389	**TTA GCA GCG GAA CAA GGA GT**	**226**
		AGC CGA GAG AAA ACA GTC CA	

### Apoptosis Assay

Cell apoptosis was determined by measuring caspase-3 levels with a Caspase-3 Colorimetric Assay Kit (R&D Systems, Minneapolis, MN) and total protein levels with a BCA (bicinchoninic acid) Protein Assay Kit (Pierce Biotechnology, Rockford, IL). Cells (2 × 10^6^) were seeded in 60-mm dishes and incubated 16 hours before being treated with kaempferol (0 or 20 μM) and cisplatin (0, 40, and 80 μM) for 2 hours. After 3 washes with cold PBS, cells were harvested in lysis buffer, and cell lysates were collected for caspase-3 assay and for the BCA assay as per instructions. Standard curves were generated by serial dilution of recombinant human caspase-3 (R&D Systems) and albumin standards (2 mg/mL), and caspase-3 levels were normalized to total protein levels for each sample. Normalized caspase-3 levels were expressed as percent of control and logged for linear regression analysis. A total of 4 independent experiments were performed for statistical analysis.

### Statistical Analysis

All data were normalized as percent of control within each experiment and data from several independent experiments were pooled to represent three biological replicates. For mRNA levels and caspase-3 levels, treatment groups with various concentrations of cisplatin and/or kaempferol were compared against control by ANOVA with Dunnett t multiple comparisons in SPSS 15.0 software (SPSS Inc, Chicago, IL). For detection of interactions between cisplatin and compounds, cell viability levels and caspase-3 levels (at logarithmic scale) were plotted against cisplatin (coded as 0, 1, 2 to represent 0, 40, and 80 μM cisplatin, respectively) with or without 20-μM test chemical (coded as 0, 1) for linear regression analysis in SPSS 15.0 software. Cisplatin, compound, and the product between cisplatin and compound were entered as 3 predictors for linear regression analysis to examine the effects of cisplatin, compound, and cisplatin-compound interaction, respectively (Y = Constant + Cisplatin + Compound + Cisplatin*Compound). An additive effect between cisplatin and compound will give two lines nearly parallel, while an interaction will have unparallel lines and a significant coefficient for the product predictor: Cisplatin*Compound. A sensitization effect can be seen when there is an interaction between cisplatin and compound and the effect of cisplatin is amplified by co-treatment with compound: two lines that depart from each other when moving toward higher cisplatin concentrations. The relationship between cMyc and CDKN1A genes' mRNA levles was evaluated by Pears' Correlation Analysis in SPSS software.

## Abbreviations

VEGF: vascular endothelial growth factor; CDKN1A: Cyclin-dependent kinase inhibitor 1A.

## Declaration of Competing interests

The authors declare that they have no competing interests.

## Authors' contributions

HL carried out the majority of experimental work. YC drafted the manuscript. All authors participated experimental design, read and approved the final manuscript.
